# Cathepsin L, a Target of Hypoxia-Inducible Factor-1-α, Is Involved in Melanosome Degradation in Melanocytes

**DOI:** 10.3390/ijms22168596

**Published:** 2021-08-10

**Authors:** Ji Young Kim, Eun Jung Lee, Yuri Ahn, Sujin Park, Yu Jeong Bae, Tae Gyun Kim, Sang Ho Oh

**Affiliations:** 1 Department of Dermatology and Cutaneous Biology Research Institute, Severance Hospital, Yonsei University College of Medicine, Seoul 03722, Korea; snyd@yuhs.ac (J.Y.K.); leeej87@yuhs.ac (E.J.L.); arashi0816@naver.com (Y.A.); parksj94@yuhs.ac (S.P.); jjuddo@yuhs.ac (Y.J.B.); tgmed83@yuhs.ac (T.G.K.); 2 Graduate School of Medical Science, Brain Korea 21 FOUR Project for Medical Science, Yonsei University College of Medicine, Seoul 03722, Korea

**Keywords:** Cathepsin L, hypoxia-inducible factor-1-alpha, autophagy, melanosome

## Abstract

Hypoxic conditions induce the activation of hypoxia-inducible factor-1α (HIF-1α) to restore the supply of oxygen to tissues and cells. Activated HIF-1α translocates into the nucleus and binds to hypoxia response elements to promote the transcription of target genes. Cathepsin L (CTSL) is a lysosomal protease that degrades cellular proteins via the endolysosomal pathway. In this study, we attempted to determine if CTSL is a hypoxia responsive target gene of HIF-1α, and decipher its role in melanocytes in association with the autophagic pathway. The results of our luciferase reporter assay showed that the expression of CTSL is transcriptionally activated through the binding of HIF1-α at its promoter. Under autophagy-inducing starvation conditions, HIF-1α and CTSL expression is highly upregulated in melan-a cells. The mature form of CTSL is closely involved in melanosome degradation through lysosomal activity upon autophagosome–lysosome fusion. The inhibition of conversion of pro-CTSL to mature CTSL leads to the accumulation of gp100 and tyrosinase in addition to microtubule-associated protein 1 light chain 3 (LC3) II, due to decreased lysosomal activity in the autophagic pathway. In conclusion, we have identified that CTSL, a novel target of HIF-1α, participates in melanosome degradation in melanocytes through lysosomal activity during autophagosome–lysosome fusion.

## 1. Introduction

Hypoxia is a state in which tissues are deprived of oxygen supply. Oxygen acts as a terminal electron acceptor in the mitochondria of cells; thus, a low supply of oxygen is closely related to the pathology of diseases such as cancer, aging, and diabetes [1]. Cells recognize changes in oxygen concentration and respond to low oxygen concentrations by activating hypoxia-inducible factor-1 (HIF-1). HIF-1 is a heterodimer consisting of HIF-1α (O2-labile) and aryl hydrocarbon receptor nuclear translocator (HIF-1β). Among the HIF-1 subunits, the status of HIF-1α determines the activity of HIF-1 and is known as a transcriptional regulator of the hypoxia-responsive genes that are related to cell proliferation, survival, death, cytoskeletal structure, and angiogenesis [2]. Under hypoxic or hypoxia mimicking conditions, accumulated HIF-1α is translocated to the nucleus and binds to hypoxia response elements (HRE) in the genome containing the sequence 5′-RCGTG-3′ (R, purine (A or G)) to activate the transcription of target genes, including vascular endothelial growth factor (VEGF), transforming growth factor-β (TGF-β), and phosphoglycerate kinase 1 (PGK1) [3,4,5]. Skin has also been reported to be a participant in response to hypoxia. Although the skin is exposed to air, the epidermal oxygen pressure remains relatively low. Previous studies have reported that HIF-1α participates in cutaneous angiogenesis, skin tumorigenesis, and microbial infection. HIF-1α induces the migration and motility of dermal fibroblasts and keratinocytes [6,7]. To understand the elaborate network of HIF-1α, the mechanism of HIF-1α and its target genes needs to be investigated.

Lysosomal proteases participate in protein hydrolysis and are related to diseases such as cancer, osteoporosis, and Alzheimer’s disease [7,8,9,10]. Among the papain family of cysteine proteinases, cathepsin B (CTSB) is reported to participate in tumor cell proliferation, angiogenesis, and invasion [11,12,13], and has been identified as a target of HIF-1α [9]. Cathepsin K (CTSK) plays a role in osteoclast-mediated bone resorption by degrading bone matrix proteins [9]. Like CTSB and CTSK, cathepsin L (CTSL) is released from the lysosome, located in endolysosomal vesicles, and acts as an endopeptidase. Based on these findings, we hypothesized that other cathepsin subtypes, such as CTSL, could be regulated by HIF-1α as CTSB. Therefore, we manually searched for hypoxia response elements (HREs) in promoters of CTSL and, as a result, we found three HREs in CTSL using ApE plasmid Editor.

CTSL is subdivided into CTSL1 and CTSL2 in humans, which are termed CTSL and CTSV, respectively, in mice [14]. The function of CTSL has been studied in various fields. CTSL is involved in disease-related cellular processes such as tumor cell migration, processing of antigenic peptides, B cell homeostasis, increased aortic diameters, and arterial wall remodeling [15,16,17]. Recently, a relationship between CTSL and coronavirus (SARS-CoV-2) disease (COVID-19) has been reported. Elevated levels of circulating CTSL in COVID-19 patients cleave the spike protein of the virus and result in increased virus entry into cells [18]. Additionally, a previous report has revealed that CTSL is required for normal hair follicle development, cycling, homeostasis of the interfollicular epidermis, and hair follicle pigmentation [19]. Tobin et al. has reported that CTSL knockout mice demonstrate abnormalities in hair canal formation, and melanosomes in the hair follicles become unstable when CTSL is absent [20]. Accordingly, CTSL is known to play an important role in skin physiology, such as hair follicles and melanocytes. Although many studies demonstrated the relationship between CTSL and cellular signaling, there is no report on the role of CTSL, regulated by HIF-1α, in melanocytes and fibroblasts. Thus, we have first shown that HIF-1α regulates CTSL expression using the melan-a cells through a luciferase gene reporter assay. In addition, HIF-1α-regulated CTSL expression in melanocytes participates in melanosome homeostasis through the lysosomal and autophagic pathways.

## 2. Results

### 2.1. Hypoxia Condition Induces the Nuclear Expression of HIF-1α Protein and CTSL Expression in Melan-a Cells

**Figure 1:**
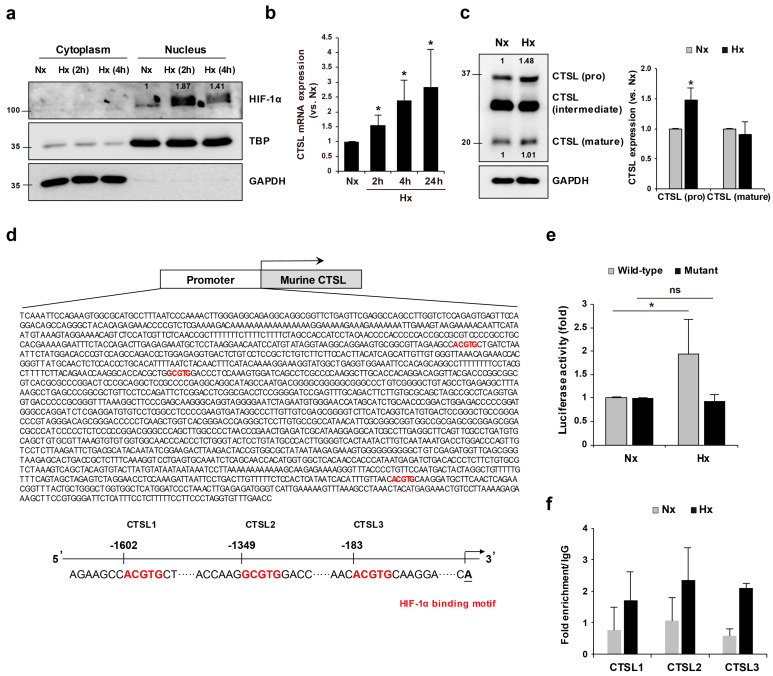
Hypoxic condition enhanced HIF-1α and CTSL activation. (**a**) Western blotting analysis of cytosolic and nuclear fractions of HIF-1α under normoxic or hypoxic conditions. mRNA (**b**) and protein (**c**) levels of CTSL were assessed in melan-a cells in hypoxia. (**d**) HREs in CTSL promoter were predicted. (**e**) Cells were transfected with luciferase reporter vectors, pGL3-Ctsl/HRE or pGL3-Ctsl/mutHRE, and luciferase activity was determined under hypoxic conditions for 4 h. (**f**) ChIP-qPCR analysis for HIF1-α occupancy on CTSL promoter in melan-a cells in normoxa or hypoxia for 4 h. Quantification of enrichment was represented as fold-enrichment relative to IgG. Results are expressed as mean ± S.D of more than three independent experiments. * *p* < 0.05. Student’s *t*-test.

The translocation of HIF-1 α into the nucleus was evaluated under hypoxia conditions. As shown in Figure 1a, hypoxia enhanced yhr nuclear fractions of HIF-1 α protein. In addition, CTSL mRNA (Figure 1b) and protein (Figure 1c) were induced by hypoxia environment. Next, we detected three candidate consensus HREs (RCGTG, R, purine (A or G)) in the murine CTSL promoter (Figure 1d) and designed a luciferase reporter construct containing murine CTSL with HREs (pGL3-*Ctsl*/HRE) and mutated HREs (pGL3-*Ctsl*/mutHRE). Luciferase assay results demonstrate that cells transfected with wild-type construct (pGL3-*Ctsl*/HRE) showed higher luciferase activity in hypoxia conditions compared to normoxia (Figure 1e). However, mutant construct (pGL3-Ctsl/mutHRE)-transfected cells did not show incremental activity under hypoxia. To confirm the interaction between HIF-1 α and CTSL promoter in melan-a cells, chromatin immunoprecipitation was performed. Since the CTSL promoter contains three candidate consensus HREs, we tested three different sites to detect interaction. Figure 1f shows that HIF-1α bound to all three sites under hypoxia conditions. Based on these results, CTSL transcription was induced by HIF-1α binding to the predicted sequence in the CTSL promoter.

### 2.2. Hypoxia Mimicking Condition by DFO Treatment Also Stimulated the Nuclear Expression of HIF-1α Protein and CTSL Expression in Melan-a Cells

Deferoxamine (DFO), an iron chelator, is known to upregulate the expression of HIF-1α by mimicking the hypoxic environment [14,21]. The findings of cell viability assays indicate that DFO treatment does not induce cytotoxicity at concentrations below 100 μM (Figure S1a). Furthermore, the DFO treatment of melan-a cells significantly increased the nuclear expression of HIF-1α protein (Figure S1b) and CTSL transcripts (Figure S1c). Moreover, the protein levels of CTSL and LC3 II were increased after DFO treatment (Figure S1d). These results indicate that DFO induces HIF-1α and CTSL in melan-a cells.

### 2.3. HIF-1α and CTSL Are Induced in Melan-a Cells under Starvation Conditions

**Figure 2:**
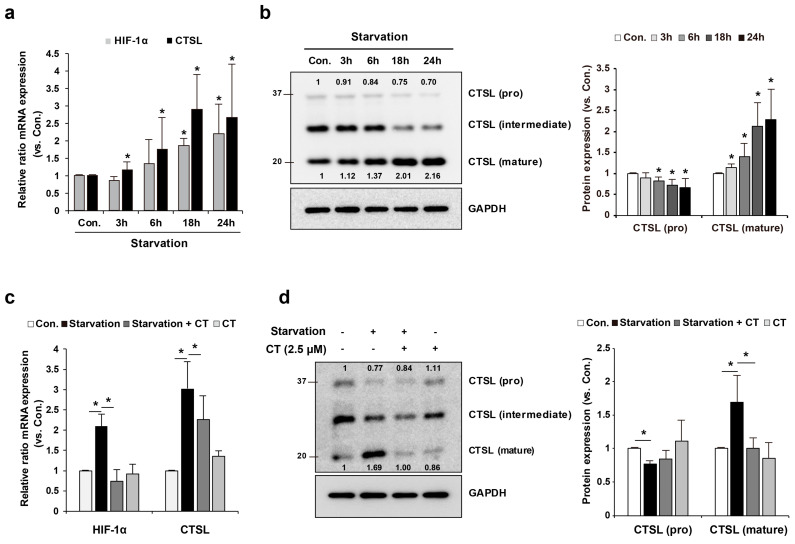
HIF-1α and CTSL induction was enhanced by starvation condition in melan-a cells. (**a**) mRNA levels of HIF-1α and CTSL were evaluated under starvation control. Moreover, (**b**) the protein level of CTSL was examined. Melan-a cells were pretreated with CT for 1 h and (**c**) mRNA levels of HIF-1α and CTSL were determined under starvation for 24 h. (**d**) Protein level of CTSL was also confirmed. Results are expressed as mean ± S.D of more than three independent experiments. * *p* < 0.05. Student’s *t*-test.

Previous studies have shown that CTSL participates in autophagy [22]. To evaluate the role of CTSL in autophagy and its association with HIF-1α in melanocytes, a starvation model was employed to induce autophagy. Melan-a melanocytes were cultured under starvation conditions, and the mRNA and protein levels of HIF-1α and CTSL were evaluated. Our results reveal that as the starvation time increased, the mRNA levels of both the molecules were also enhanced (Figure 2a). In addition, the conversion of pro-CTSL to mature CTSL was significantly increased with starvation, as shown by Western blotting analysis (Figure 2b). To verify the effect of HIF-1α on CTSL expression, melan-a cells were pre-treated with cryptotashinone (CT), an HIF-1α inhibitor, and cells were under starvation. The augmented expressions of HIF-1α and CTSL at the transcript (Figure 2c) and mature CTSL at protein (Figure 2d) levels under starvation conditions were attenuated in the presence of CT. These results imply that HIF-1α is closely related to CTSL expression in melan-a melanocytes.

### 2.4. CTSL Is Associated with Melanosome Degradation through Autophagy in Melan-a Cells

**Figure 3:**
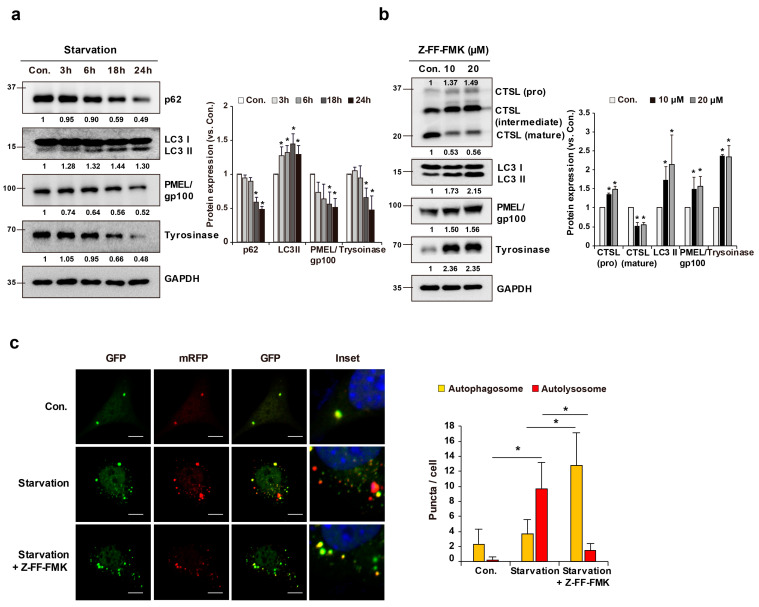
CTSL is associated with melanosome degradation through autophagy in melan-a cells. (**a**) Protein levels of p62, LC3, PMEL/gp100, and tyrosinase were examined under starvation conditions. (**b**) Melan-a cells were treated with Z-FF-FMK for 1 h and protein levels of CTSL, LC3, PMEL/gp100, and tyrosinase were evaluated. (**c**) After Z-FF-FMK pre-treatment and starvation for 8 h, tandem fluorescent-tagged LC3 (mRFP-GFP-LC3) was observed by confocal microscopy. Yellow (autophagosome) and red (autolysosome). Scale bar: 10 μm. Results are expressed as mean ± S.D of more than three independent experiments. * *p* < 0.05. Student’s *t*-test.

Analysis of melan–a cells subjected to starvation conditions has shown that starvation induces the conversion of LC3 I to LC3 II, a central protein in autophagy, as well as a decrease in the p62 levels in these cells. In addition, starvation decreased the protein levels of gp100 and tyrosinase in melan-a cells (Figure 3a). To identify the role of CTSL in melanocytes in association with autophagy and melanosome regulation, the melan-a cells were treated with Z-FF-FMK, an inhibitor that prevents the conversion of pro-CTSL into mature CTSL. As a result, the conversion of CTSL from the pro- to the mature form was blocked by treatment with Z-FF-FMK. Furthermore, along with increased expression of LC3 II, increased expression of gp100 was observed in melan-a cells upon treatment with Z-FF-FMK (Figure 3b). Next, using tandem fluorescent-tagged LC3 (mRFP-GFP-LC3) plasmid, we examined the role of the mature form of CTSL in the starvation-induced autophagy pathway in melan-a cells. Our results show that starvation induced autophagic flux, based on the finding that the numbers of yellow and red puncta were increased in melan-a cells. In accordance with a previous report, an inhibitor of CTSL conversion to the mature form decreased the number of red puncta, suggesting that the accumulation of pro-CTSL in lysosomes led to incomplete autophagy by lysosomal dysfunction (Figure 3c) [23]. These results suggest that the mature form of CTSL is important in the regulation of autophagosome-lysosome fusion for the completion of autophagy.

**Figure 4:**
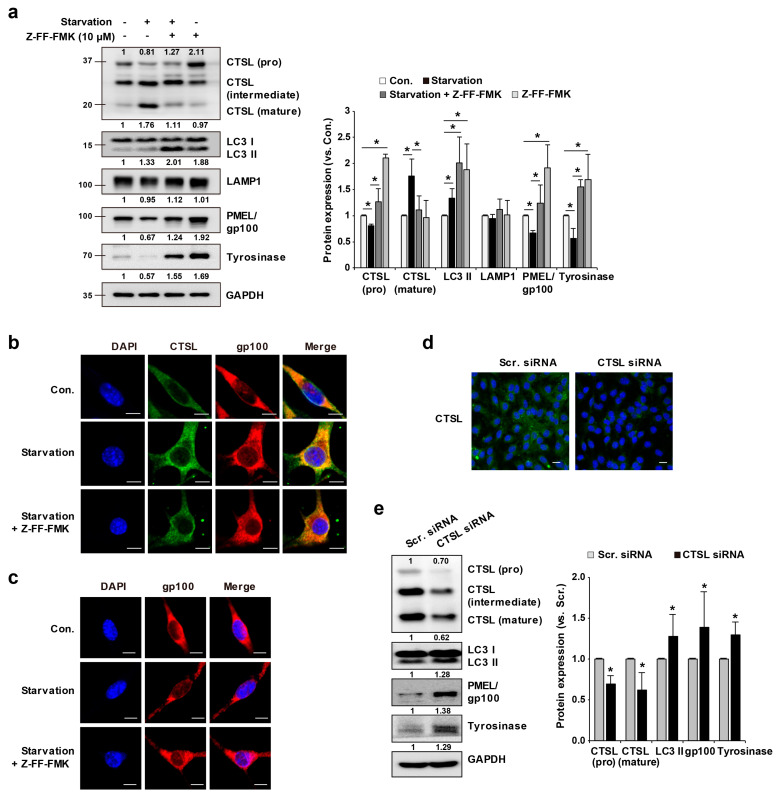
Inhibition of CTSL activity led to accumulation of melanosome-related molecules in melan-a cells. (**a**) After Z-FF-FMK pre-treatment and starvation for 24 h, CTSL, LC3, PMEL/gp100, and tyrosinase protein expressions were determined. (**b**) CTSL and gp100 expressions were observed by confocal microscopy after Z-FF-FMK pre-treatment and starvation for 4 h. Scale bar: 10 μm. (**c**) gp100 expression was observed by confocal microscopy after Z-FF-FMK pre-treatment and starvation for 24 h. Scale bar: 10 μm. (**d**) Confocal image of melan-a cells after scramble (scr) siRNA or CTSL siRNA treatment for 48 h. Scale bar: 20 μm. (**e**) CTSL, LC3, PMEL/gp100, and tyrosinase protein expressions were evaluated after scramble (scr) siRNA or CTSL siRNA treatment for 48 h. Results are expressed as mean ± S.D of more than three independent experiments. * *p* < 0.05. Student’s *t*-test.

We next evaluated the role of CTSL in melanosome degradation in association with the autophagic pathway. Starvation increased the expression of LC3 II, and the conversion of pro-CTSL to mature CTSL. When Z-FF-FMK was used, a decrease in the mature form of CTSL and accumulation of LC3 II were observed, possibly due to decreased lysosomal activity in the autophagic pathway. In addition, starvation decreased the expression of gp100 and tyrosinase, while inhibiting the conversion of pro-CTSL to mature CTSL, results in the accumulation of gp100 and tyrosinase (Figure 4a). Additionally, confocal images show that CTSL expression after 4 h-starvation was colocalized with gp100 whereas Z-FF-FMK pre-treatment attenuated colocalized CTSL and gp100 induced by starvation (Figure 4b). After 24 h-starvation, gp100 expression was significantly decreased compared to control. When Z-FF-FMK was pre-treated, gp100 expression was not reduced even under starvation (Figure 4c). These findings imply that starvation induced melanosome degradation in melanocytes by inducing autophagy along with the increased conversion of CTSL to the mature form. If conversion of CTSL into the mature form is blocked, melanosome degradation by starvation-induced autophagy is inhibited, along with the accumulation of LC3 II. Moreover, we treated CTSL siRNA to melan-a cells to confirm the role of CTSL in lysosomal degradation of LC3II at the lysosomal level. CTSL-knockdown cells showed significantly higher expressions of LC3II and melanosome markers (gp100 and tyrosinase) compared to scramble siRNA-treated melan-a cells (Figure 4d,e). This result confirms that CTSL is involved in autophagy and melanosome degradation.

### 2.5. The Regulation of CTSL Transcription by HIF-1α Was Also Detected in NIH-3T3 Cells

The association of HIF-1α with CTSL observed in the melan-a cells was further evaluated in the NIH-3T3 cells, mouse fibroblast cell line. First, DFO was applied to the NIH-3T3 fibroblast cell line to determine cell viability using the MTT assay (Figure S2a). We then evaluated the translocation of HIF-1 α into the nucleus using Western blot analysis. Our results showed the clear enhancement of nuclear fractions of HIF-1α protein in the cells treated with DFO (50, 100, and 200 μM) (Figure S2b). mRNA levels of *CTSL* were increased upon treatment with DFO at the indicated concentrations (Figure S2c). Additionally, DFO treatment significantly enhanced the luciferase activity in pGL3-*Ctsl*/HRE-transfected NIH-3T3 cells, whereas luciferase activity remained unchanged in pGL3-*Ctsl*/mutHRE-transfected cells (Figure S2d). These findings imply that the promoter sequence encompassing the HIF-1α binding site is closely related to *Ctsl* gene transcription, indicating that HIF-1α controls the transcription of the *Ctsl* gene.

mRNA expressions of *HIF-1α* and *CTSL* significantly were increased after 24 h treatment with DFO (Figure S3a). In addition, the protein levels of HIF-1α and CTSL (proform) were also induced with an increased incubation time of DFO treatment (Figure S3b). To verify the effect of HIF-1α on CTSL expression, the NIH-3T3 cells were treated with CT prior to DFO treatment. Our results indicated that DFO treatment activated the expression of HIF-1α and CTSL at the transcript (Figure S3c) and protein levels (Figure S3d), whereas the expression of HIF-1α and CTSL, which were induced by DFO treatment, were abolished in CT-pre-incubated NIH-3T3 cells.

To determine the role of CTSL in autophagy and apoptosis, NIH-3T3 cells were treated with Z-FF-FMK and cell viability was assessed. Adding Z-FF-FMK to DFO-treated cells results in remarkably decreased cell viability as compared to DFO-only treated cells, suggesting that the conversion of CTSL to the mature form could be involved in the regulation of the viability of cells (Figure S4a). Subsequently, we examined the changes in the expression of CTSL, LC3 (Figure S4b) and cleaved caspase-3 (Figure S4c), an apoptosis-related molecule, following treatment with DFO and Z-FF-FMK. Our data revealed that Z-FF-FMK pretreatment of DFO-treated cells shows significantly higher levels of LC3 II conversion and cleaved caspase-3, while no increase was observed in the mature form of CTSL, as compared to DFO-only treated cells. These results suggest that the mature form of CTSL might play an important role in the regulation of cell survival and the autophagy pathway.

## 3. Discussion

The present study has demonstrated that CTSL transcription is regulated by HIF-1α expression. Moreover, the HIF-1α induced-expression of CTSL is involved in lysosomal dynamics that are associated with the autophagy pathway and melanosome degradation in melanocytes. Previously, CTSB, another cathepsin molecule, was studied as a target of HIF-1α in HepG2 cells [12]. However, there is no report on whether CTSL is regulated by HIF-1α. In our study, we have shown that hypoxia or hypoxia-mimicking conditions by DFO treatment trigger the expression of HIF-1α and CTSL in melan-a cells and NIH-3T3 cells. In particular, the role of HIF-1α in the regulation of CTSL expression was confirmed via a luciferase gene reporter assay. Furthermore, the regulation of HIF-1α-induced CTSL expression was verified by experiments involving the use of an HIF-1α inhibitor.

HIF-1α is important in skin physiology and pathophysiology because skin, under a relatively hypoxic environment, favors increased expression of HIF-1α, which regulates a variety of biological processes in the skin [24]. Under normoxic conditions, prolyl-4-hydroxylases (PHDs) hydroxylate HIF-1α and bind with von Hippel–Lindau protein (pVHL), which is an E3 ubiquitin ligase. Then, hydroxylated HIF-1α is processed to proteosomal degradation. Under hypoxic conditions, PHDs are inactivated, and HIF-1α is translocated to the nucleus, where it binds to the HRE of target genes promoters to induce transcription [25,26]. HIF-1α is found to regulate the expression of hundreds of genes involved in many physiological functions, including angiogenesis, cell survival, migration, metastasis, and glycolysis [27]. In the skin, HIF-1α plays an important role in the adhesion and migration of keratinocytes. Further, HIF-1α knockdown in keratinocytes leads to a decreased expression of laminin-332, α6 integrin, and β1 integrin, which induces dysregulated cell proliferation, colony-forming efficiency, and cell cycle arrest [28].

In our study, we have reported that CTSL transcription is regulated by the binding of HIF-1α to the HREs present on the CTSL promoter. Our promoter analysis has revealed three putative candidate consensus HREs in the CTSL promoter, which led to the hypothesis that CTSL could be one of the HIF-1α target genes. Based on the outcomes of the luciferase gene reporter assay, it was evidently shown that the putative HREs in the CTSL promoter regulate the transcription of CTSL in an HIF-1α dependent manner. Moreover, HIF-1α-mediated CTSL expression was confirmed by experiments using an HIF-1α inhibitor. CTSL degrades cellular proteins through the endolysosomal pathway. Moreover, CTSL participates in the autophagic pathway by degrading autophagosome markers LC3 II and GABARAP-II [29]. Our results have shown that starvation induces the conversion of LC3 I to LC3 II, along with an increased expression of the mature form of CTSL. Moreover, an inhibitor that prevents the conversion of CTSL to the mature form induces the accumulation of LC3 II by blocking LC3 II degradation through the progression from autophagosomes to autolysosomes in the autophagic pathway. A previous study also revealed that the inhibition of CTSL activity induces impaired autophagy, which is mediated by lysosomal dysfunction [23]. In accordance with the findings of a previous report, the accumulation of pro-CTSL without conversion to the mature form inhibits the lysosomal degradation, leading to lysosomal dysfunction and abnormal autophagic pathways. Therefore, CTSL activity might be important for the completion of autophagy by autophagosome–lysosome fusion.

Along with the effect of CTSL on autophagic signaling, HIF-1α is also known to be associated with autophagy signaling. Autophagy is a mechanism by which cytoplasmic materials, such as damaged or unnecessary organelles, are processed by double membrane vesicles called autophagosomes, and delivered to the lysosomes. This signaling pathway is responsible for removing and recycling the dysfunctional cellular materials [30]. It has been reported that autophagy is activated under hypoxic conditions, leading to the induction of HIF-1α. This process protects the cells from hypoxic conditions, facilitating oxygen homeostasis [31]. Our results also show that HIF-1α induction promotes CTSL expression and leads to autophagy. Taken together with previous studies, the findings of our study reinforce that HIF-1α under hypoxic conditions is closely related to autophagic signaling.

We have also validated the effect of CTSL on melanosome homeostasis. Melanosomes are important organelles for melanin production and include tyrosinase and tyrosinase-related proteins, which play critical roles in melanogenesis [32,33]. Many studies have demonstrated that autophagy participates in the MITF-target gene regulation, melanosome biogenesis, and melanosome degradation [34,35,36]. In particular, it has been reported that melanosome degradation in keratinocytes, as well as melanocytes, can be controlled by the regulation of autophagic activity [37,38]. Moreover, melanosomes may become unstable when CTSL is absent because melanosomes exhibit lysosomal characteristics [20,33]. Our observations have also confirmed that CTSL-induced autophagic signaling results in reduced gp100 expression, and the inhibition of CTSL maturation stimulates the accumulation of LC3 II without further progression from autophagosomes to autolysosomes by the autophagosome–lysosome fusion. In addition, melanosomes are also accumulated through the impairment of autophagy completion by lysosomal dysfunction. According to previous studies, cathepsin V, another cathepsin molecule, is reported to play a vital role in pigmentation. The expression of cathepsin V, known as CTSL2, has been reported to be higher in keratinocytes from light skin than in those from dark skin, even though there was no difference in the expression of various subtypes of cathepsin such as CTSL, CTSL2, and CSTB in melanocytes between light and dark skins [39]. Moreover, CTSV expression has been reported to be involved in melanosome degradation [40]. Therefore, CTSL as a lysosomal enzyme, which was examined in this study in association with HIF-1α, might also play a crucial role in the regulation of melanosomal degradation through the autophagy pathway.

Taken together, the current study highlights that *CTSL* is a novel target gene of HIF-1α and plays a pivotal role in melanosome homeostasis through the autophagic pathway. We believe that the findings from this study provide a useful resource for studying the HIF-1α-CTSL axis and its involvement in the autophagy pathway in melanocyte biology.

## 4. Materials and Methods

### 4.1. Cell Cultures

NIH-3T3 fibroblasts, derived from mouse embryonic fibroblast cells, were cultured in Dulbecco’s modified Eagle’s medium (DMEM, Welgene, Daegu, Republic of Korea) with 10% fetal bovine serum (FBS, Welgene) and 1% penicillin-streptomycin (Welgene). Melan-a, a highly pigmented non-tumorigenic mouse melanocytic cell line, was kindly provided by Prof. Dorothy C. Bennett (St George’s Hospital Medical School, London, UK). The cells were maintained in RPMI 1640 growth medium (Welgene), supplemented with 10% FBS, 1% penicillin-streptomycin, and 200 nM 12-*O*-tetradecanoylphorbol-13-acetate (TPA; Sigma-Aldrich, St Louis, MO, USA). Cells were maintained at 37 °C in a humidified 5% CO_2_ incubator. For hypoxia, cells were grown in a hypoxia incubator at 37 °C in 1% O_2_ and 5% CO_2_.

### 4.2. Reagents

Deferoxamine mesylate salt (DFO), Z-FF-FMK, and cryptotanshinone (CT) were purchased from Sigma-Aldrich (St Louis, MO, USA).

### 4.3. Dual Luciferase Assay

The consensus sequence of the three HRE sites in the −2000 bp fragment of the mouse CTSL promoter were mutated by substituting CG or GC with AA. The DNA fragment was cloned into vector pGL3 to generate pGL3-Ctsl/HRE or pGL3-Ctsl/mutHRE. Melan-a and NIH-3T3 cells were seeded onto 96-well plates at a density of 5 × 10^3^ cells/well, and allowed to attach for 24 h. Next, cells were transfected with pGL3-HRE-luciferase construct (pGL3-Ctsl/HRE or pGL3-Ctsl/mutHRE). After transfection, cells were treated with DFO or under hypoxia condition before reporter assay. Luciferase assays were performed using the Dual-Glo Luciferase Assay System (Promega, Madison, WI, USA), according to the manufacturer’s instructions. Luminescence was measured using a microplate reader (Centro XS LB 960, Berthold, Freiburg, Germany).

### 4.4. Chromatin Immunoprecipitation Assay

Chromatin immunoprecipitation was carried out with Pierce Agarose ChIP Kit (Thermo Scientific, Waltham, MA, USA) according to the manufacturer’s protocol. Immunoprecipitation was performed using HIF1α-specific antibodiy (Invitrogen, Carlsbad, CA, USA). The eluted DNA was used as a template for quantitative PCR (qPCR) using primers specific for the CTSL promoter, CTSL1 (forward: 5′-GAAATTCCACAGCAGGCCTT-3′, reverse: 5′-CTGTGGTGCAAGCCTTGG-3′), CTSL2 (forward: 5′-TCCATGTATAGGTAAGGCAGGA-3′, reverse: 5′-AAGACAGAGCGGAGGACAGA-3′), and CTSL3 (forward: 5′-CCTGTTCCAATGACTACTAGGC-3′, reverse: 5′-AGCCCAGCAGTAAACCGTT-3′).

### 4.5. MTT Assay

Cell viability was estimated by colorimetric assay, using 3-(4,5-dimethylthiazol-2-yl)-2,5-diphenyltetrazolium bromide (MTT; Sigma-Aldrich) for incubation (4 h, 37 °C). The supernatant was discarded and replaced with dimethyl sulfoxide (DMSO) to dissolve cytoplasmic formazan crystals. Absorbance was measured at 570 nm using a microplate reader (SpectraMax 340; Molecular Devices LLC, Sunnyvale, CA, USA).

### 4.6. Quantitative Real-Time RT-PCR

Total cellular RNA was isolated using an RNeasy Plus Mini Kit (QIAGEN, Valencia, USA). cDNA was generated from 0.5 µg total RNA using a kit (PrimeScript 1st strand cDNA Synthesis Kit; Takara Bio Inc., Japan) according to the manufacturer’s protocol. The resulting cDNA was used for quantitative PCR amplification and specific sequence detection with an Applied Biosystems Step One Real-Time PCR System (Life Technologies Japan Ltd., Japan). The PCR reaction was performed using Power SYBR^®^ Green PCR Master Mix (Applied Biosystems, Foster City, USA). The relative expression of beta actin was used to standardize the reaction. The following primer pairs, specific to HIF1α, cathepsin L, and beta actin, were used to amplify cDNA: mouse HIF1α: forward (5′-ACCTTCATCGGAAACTCCAAAG-3′), reverse (5′-CTGTTAGGCTGGGAAAAGTTAGG-3′); mouse cathepsin L: forward (5′-ATCAAACCTTTAGTGCAGAGTGG-3′), reverse (5′-CTGTATTCCCCGTTGTGTAGC-3′); mouse beta actin: forward (5′-GGCTGTATTCCCCTCCATCG-3′), reverse (5′-CCAGTTGGTAACAATGCCATGT-3′). For each sample, real-time PCR was performed in triplicate.

### 4.7. Cell Starvation

Cells were washed three times with pre-warmed PBS, then incubated in starvation medium (RPMI 1640 growth medium with 1% penicillin-streptomycin) at 37 °C under 5% CO_2_.

### 4.8. Western Blotting

Whole-cell extracts were prepared in RIPA buffer (Tris-buffered saline, 0.5% deoxycholate, 0.1% SDS, 1% Triton X-100) containing a protease inhibitor cocktail (GenDEPOT, Barker TX, USA). The cytoplasmic and nuclear fractions of cells were obtained using the NE-Per^®^ reagents (Thermo scientific, Bremen, Germany), according to the manufacturer’s instructions. Proteins isolated from cells were separated by SDS-PAGE. The separated proteins were then transferred to nitrocellulose membranes according to standard procedures. The membranes were blocked with 5% *v*/*v* skim milk in a TBS-T buffer (TBS with 0.1% *w*/*v* Tween-20) and reacted with primary antibodies overnight at 4 °C. Primary antibodies against HIF1α (Cayman Chemical, Ann Arbor, MI, USA), cathepsin L (R&D, Minneapolis, USA), cleaved caspase3, PMEL/gp100 (abcam, Cambridge, UK), Tyrosinase (Santa Cruz Biotechnology, Dallas, TX, USA), LC3 (Novus Biologicals, Littleton, CO, USA), p62 (Cell signaling Technology, Danvers, MA, USA), and LAMP1 (abcam) were used at a 1:1000 dilution. GAPDH (Santa Cruz Biotechnology, Santa Cruz, CA, USA) and TBP (abcam) were used as a control for protein loading. The membranes were then washed three times for 10 min with the TBS-T buffer and incubated for 1 h with 1% skim milk in TBS-T buffer containing horseradish peroxidase-conjugated secondary antibodies (diluted 1:3,000). The proteins were visualized using an ECL system (Thermo Scientific, Franklin, MA, USA). Signal density was quantified using Image J software (US National Institutes of Health, Bethesda, MD, USA).

### 4.9. Transfection and Plasmids

The mRFP-eGFP-LC3 construct was kindly provided by Dr. Tamotsu Yoshimori (Osaka University, Osaka, Japan). Cells were seeded into a chamber slide and cultured overnight. Melan-a cells were transfected with ptfLC3-expressing plasmid using TransIT-2020 Transfection reagent according to the manufacturer’s protocol (Mirus, Madison, WI, USA). The cells were then washed in PBS and fixed in freshly prepared 4% paraformaldehyde. After three washes in PBS, the cells were mounted with DAPI. Autophagic flux was determined based on patterns of GFP and RFP puncta under a Zeiss confocal lase-scanning microscope.

### 4.10. Confocal Microscopy

Melan-a cells were washed in PBS and fixed (15 min) in 4% paraformaldehyde. Samples were then washed in PBST (PBS with 0.1% Tween-20) and incubated overnight (4 °C) in anti-cathepsin L (R&D), or gp100 (abcam), followed by three washings in PBST and incubation (1 h) with the appropriate secondary antibody. All samples were mounted in 4′,6-diamidino-2-phenylindole solution (DAPI; Vector Laboratories Inc., Burlingame, CA, USA.). Slides were evaluated with confocal microscopy (LSM 700; Carl Zeiss, Oberkochen, Germany).

## 5. Statistical Analysis

All graphs represent the mean ± standard deviation (SD) of experiments performed more than triplicate. Data were compared using two-tailed Student’s *t*-test. Standard software (SPSS; SPSS Inc., Chicago, IL, USA) was employed, with the cutoff for statistical significance set at *p* < 0.05 (denoted by * in the figures).

## Data Availability

Not applicable.

## Supplementary Materials

The following are available online at https://www.mdpi.com/article/10.3390/ijms22168596/s1, Figure S1: HIF-1α and CTSL induction was enhanced by DFO treatment in melan-a cells, Figure S2: Hypoxic-mimic condition by DFO promoted HIF-1α and CTSL activation in NIH-3T3 cells, Figure S3: HIF-1α induction regulated CTSL expression, Figure S4: CTSL is involved in cell associated autophagy induction.

## Abbreviations

HIF-1α	Hypoxia-inducible factor-1α
CTSL	Cathepsin L
HRE	Hypoxia response elements
VEGF	Vascular endothelial growth factor
TGF-β	Transforming growth factor-β
PGK1	Phosphoglycerate kinase 1
CTSB	Cathepsin B
CTSK	Cathepsin K
COVID-19	Coronavirus (SARS-CoV-2) disease
DFO	Deferoxamine
CT	Cryptotashinone
LC3	Microtubule-associated protein 1 light chain 3
PHDs	Prolyl-4-hydroxylases
pVHL	von Hippel–Lindau protein
