# Supplementary material for: Cathepsin L, a Target of Hypoxia-Inducible Factor-1-α, Is Involved in Melanosome Degradation in Melanocytes
Source: Int J Mol Sci. 2021 Aug 10;22(16):8596. doi: 10.3390/ijms22168596 (PMC8395286; doi:10.3390/ijms22168596)
Supplement: Supplementary file 1 [file ijms-22-08596-s001.zip › ijms-1223419-supplementary.pdf]

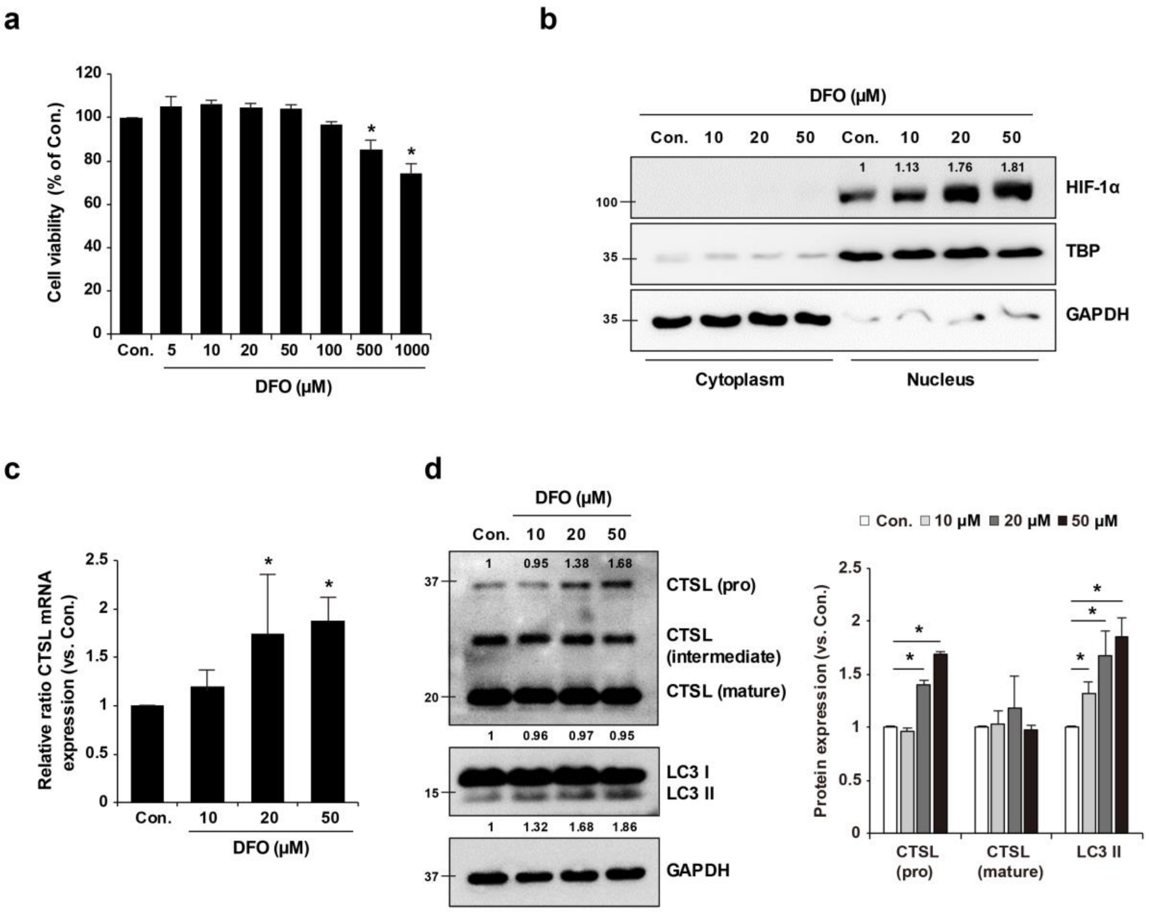

Figure S1. HIF-1α and CTSL induction was enhanced by DFO treatment in melan-a cells. Melan-a cells were treated with DFO for 24 h and (**a**) cell viability test was assessed. (**b**) Nuclear translocation of HIF-1α, (**c**) mRNA level of CTSL, and (**d**) protein level of CTSL and LC3 were evaluated. . Results are expressed as mean ± S.D of more than three independent experiments. *p < 0.05. Student's t-test.

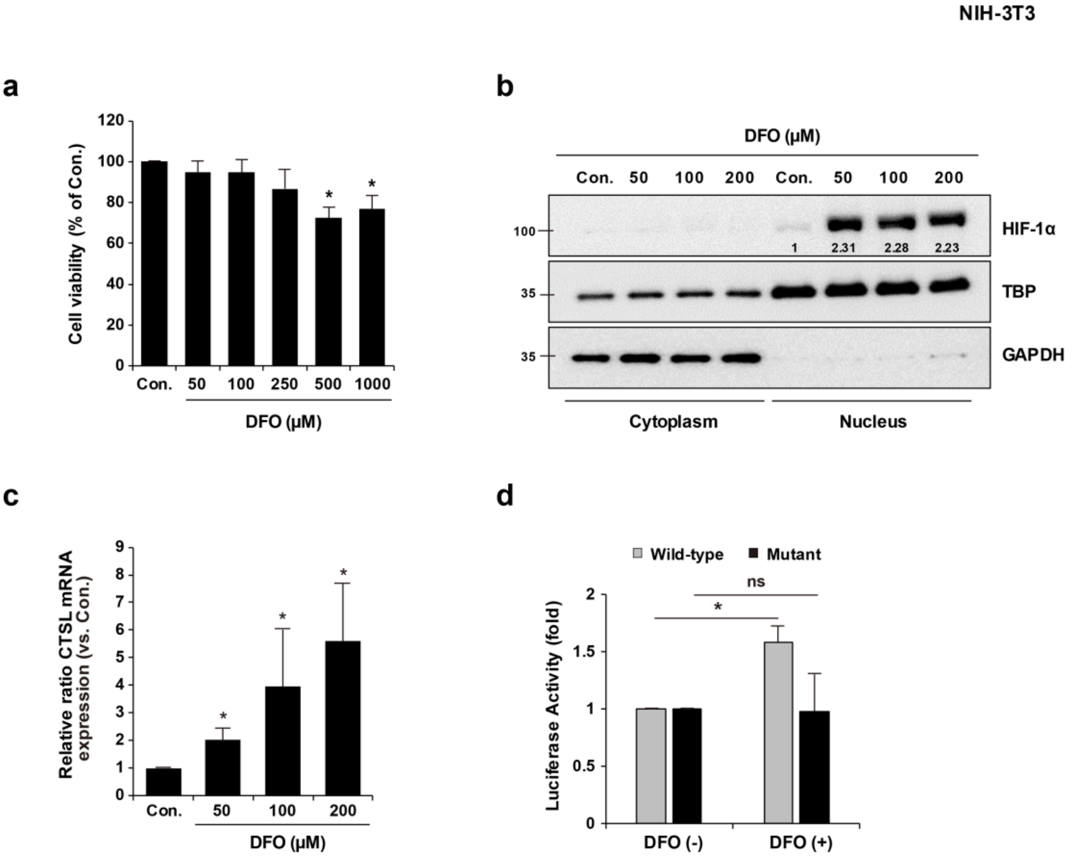

Figure S2. **Hypoxic-mimic condition by DFO promoted HIF-1α and CTSL activation in NIH-3T3 cells.** (**a**) The MTT assay was used to determine NIH-3T3 cell viability at the indicated concentrations after 24 h treatment with DFO. (**b**) Western blotting analysis of cytosolic and nuclear fractions of HIF-1α after DFO treatment. (**c**) mRNA levels of CTSL were assessed in NIH-3T3 cells treated with DFO. (**d**) Cells were transfected with luciferase reporter vectors, pGL3-Ctsl/HRE or pGL3-Ctsl/mutHRE, and luciferase activity was determined after DFO treatment. Results are expressed as mean ± S.D of more than three independent experiments. *$p < 0.05$. Student's t-test.

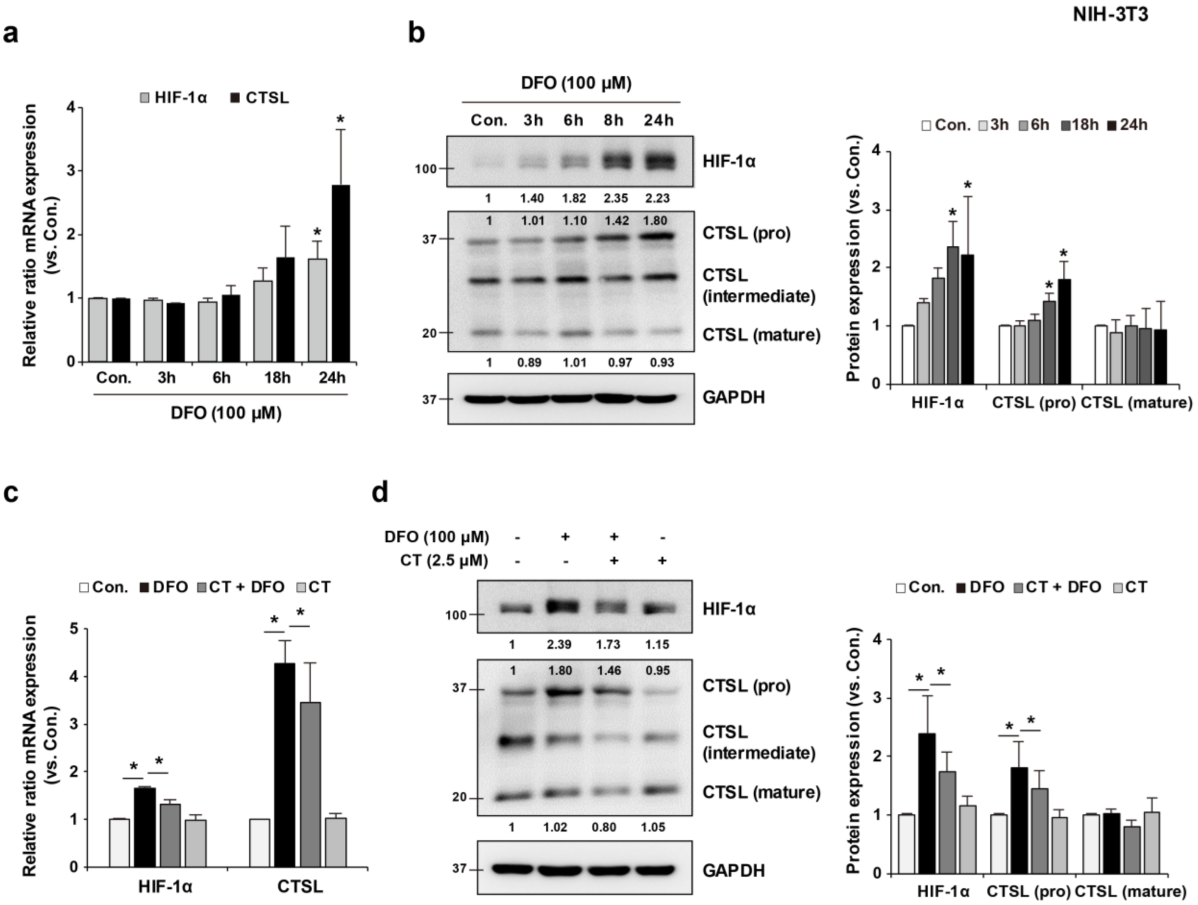

Figure S3. **HIF-1α induction regulated CTSL expression.** NIH-3T3 cells were treated with DFO. (**a**) mRNA and (**b**) protein levels of HIF-1α and CTSL were assessed. Cells were pre-treated with CT for 1 h and incubated with DFO for 24 h. (**c**) mRNA and (**d**) protein levels of HIF-1α and CTSL were evaluated. Results are expressed as mean ± S.D of more than three independent experiments. *$p < 0.05$. Student's t-test.

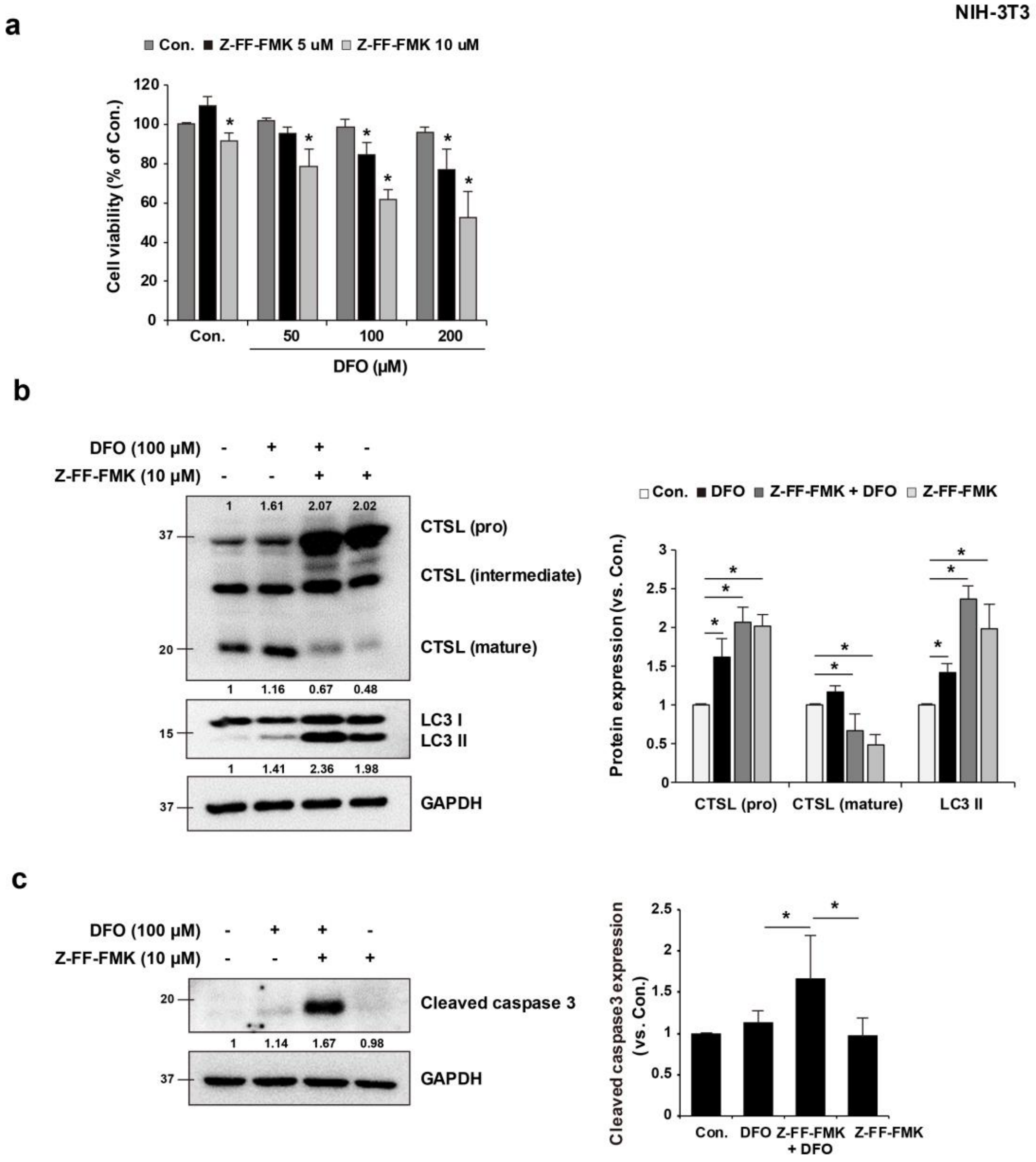

Figure S4. CTSL is involved in cell associated autophagy induction. NIH-3TS cells were pretreated with Z-FF-FMK for 1 h and incubated with DFO for 24 h. (**a**) Cell viability and protein levels of (**b**) CTSL, LC3, and (**c**) cleaved caspase 3. Results are expressed as mean ± S.D of more than three independent experiments. $*p < 0.05$. Student's t-test.
